# Assimilation and contrast effects in suboptimal affective priming paradigm

**DOI:** 10.3389/fpsyg.2014.00498

**Published:** 2014-05-26

**Authors:** Dorota Kobylińska, Dorota Karwowska

**Affiliations:** Department of Personality Psychology, Faculty of Psychology, University of WarsawWarsaw, Poland

**Keywords:** suboptimal affective priming, assimilation effect, contrast effect, emotion regulation

## Suboptimal affective priming paradigm

Contemporary psychology does not question the existence of psychological processes that operate outside human conscious awareness, such as implicit stereotypes (Greenwald and Banaji, 1995; Nosek et al., 2009), implicit attitudes (Greenwald and Banaji, 1995), automatically activated goals or norms (Bargh, 1997; Bargh and Chartrand, 2000; Bargh et al., 2001; Dijksterhuis et al., 2005), and implicit primary affect (Zajonc, 1980, 2004; Murphy and Zajonc, 1993; Murphy et al., 1995; Jarymowicz and Kobylińska, 2005; Winkielman et al., 2005; Kobylińska and Karwowska, 2007). Most data showing implicit affective stimuli influence on evaluative judgments, come from experiments conducted in an affective priming paradigm. The paradigm was introduced by Murphy and Zajonc (1993) who presented evidence for the existence of unconscious primary affect (Zajonc, 1980) and its influence on cognition. In their original experiments they presented neutral target stimuli (for example Chinese ideographs), which were preceded by either 1-s or 4-ms exposures of photographs of faces expressing either positive or negative emotions. The results showed that suboptimal (4 ms) affective primes induced affect that influenced evaluations of the neutral targets. Ideographs primed by negative facial expressions were judged more negatively than those primed by positive ones. Interestingly, neither the facial expression nor even the presence of any image was accessible to the participants' awareness. In contrast to suboptimal affective primes, both optimal affective primes (exposed for 1 s) and affectively neutral suboptimal primes (e.g., geometric figures of different shapes) failed to influence the participants' judgments about targets.

This was the first effect observed in affective priming research. However, it turned out in later studies that the reverse effect is also possible (Glaser and Banaji, 1999; Ohme et al., 1999). The first effect, when judgments about neutral stimuli are consistent with the valance affective primes, is referred to as the assimilation effect. The reverse effect, when targets primed negatively are evaluated more positively than those primed positively (thus inconsistently with priming valance), is called the contrast effect. It turned out that which of the two effects is obtained is not a matter of chance but is related to important psychological and neurobiological processes occurring at the time when affect is elicited.

Both effects are quite simple and both proof that judgments may be influenced by stimuli presented outside conscious awareness. Accordingly, in this opinion article, we will explore the psychological mechanism underlying the effects of assimilation and contrast.

## Evidence for obtaining assimilation or contrast effects in priming research

Traditionally in research applying explicit semantic priming, the contrast effect was explained as coming from the fact that participants were aware of priming manipulation or of the influence of priming on the target task (Lombardi et al., 1987; Newman and Uleman, 1990; Strack et al., 1993) or from engaging additional cognitive resources and effort to resist the primary reaction induced by priming. This primary reaction is rather based on assimilation to priming (Martin et al., 1990), while the contrast effects is usually described as resulting from more complex information processing that requires, at least, elementary processes of comparisons (Lombardi et al., 1987; Martin et al., 1990; Ric and Niedenthal, 2007). However, when contrast effect is obtained in suboptimal affective priming procedure, it cannot be explained by participants' awareness.

Glaser and Banaji (1999) found assimilation and contrast effects in their experiment on implicit semantic priming. They showed that priming words that were salient in their affective meaning produced contrast effect, whereas affectively mild words produced assimilation effect. Such results confirmed the earlier results of Herr et al. (1983). Similar pattern was later obtained in advertising studies (Vianello et al., 2009).

Ohme et al. (1999) obtained contrast effect in three experiments in suboptimal affective priming paradigm in which they used Chinese hexagrams (instead of ideographs) as target stimuli. This change required different instruction for participants. Instead of evaluating the attractiveness of targets, participants had to decide (using 5-point Likert scale) if the hexagrams represent chaos and conflict or order and harmony. Those concepts are related to negativity and positivity: chaos and conflict is associated with decay whereas order and harmony with tranquillity and peace. In all three experiments contrast effect was registered: hexagrams primed negatively were evaluated higher (more positively) then those primed positively and not primed affectively. In discussion of their results Ohme and others argued that the contrast effects were obtained because of (1) more abstract instruction, potentially engaging information processing related to the functions of the left cerebral hemisphere and (2) using hexagrams that seem to activate more analytical information processing then ideographs.

Further evidence comes from experiments by Kobylińska (2001, 2007). She based her hypotheses on the following reasoning. Some researchers indicated that in conditions of implicit affective priming the contrast effect, in comparison to the assimilation effect, results from slightly larger contribution of cognitive processes in the evaluation process (Dijksterhuis et al., 1998; Kolańczyk et al., 2001, 2007; Kolańczyk and Pawłowska-Fusiara, 2002; Mussweiler and Damisch, 2008). Contrast seems to require more detailed processing of affective information by neural system, since it appears not as a direct consequence of priming but only after employing some other parallel competing process. According to neurophysiological findings (Libet, 1996), the stronger a stimulus, the more complex neural structures participate in its processing and the more complex reactions are produced. By stronger stimulation we may understand more salient affective stimuli or just stimuli lasting longer. Zajonc (2004) claims that when the exposure of affective stimulus is lengthened, subject gains access to more information about the stimulus.

In several experiments Kobylińska (and later Kobylińska and Karwowska) used different exposure durations of affective primes as operationalization of salience of affective stimulation. Confirming the predictions, the results showed assimilation effect when the exposure duration of primes was shorter—4 ms—and the contrast effect, with the longer duration of 16 ms (Kobylińska, 2001, 2007; Kobylińska and Karwowska, 2007). The results indicate that influence of the nonconscious primary affect may be bidirectional (assimilation or contrast effects) and the conscious perception of the affective stimulus (or its influence) is not necessary for that. The lengthening of the exposure duration of affective stimuli from 4 to 16 ms does not make the stimulus accessible to consciousness. However, the influence of affect elicited by stimuli exposed for 4 or 16 ms was different, in fact it was just the reverse.

## Contrast effect as a result of implicit emotion regulation

According to Glaser and Banaji (1999) the contrast effects obtained in nonconscious affective priming research may result from spontaneous application of autocorrection process that corrects the primary, direct assimilative effects of affective primes. It can be understood as a very basic process of automatic control (Kolańczyk et al., 2001, 2007) aimed at reducing the nonconscious influence of affective primes biasing evaluation of neutral objects. This process occurs without participants' awareness, and that is why instead of resulting in neutral evaluations it results in certain overcompensation. Glaser and Kihlstrom (2005) describe many studies on different implicit phenomena (for example attitudes or stereotypes) in which contrast effects were obtained. They suggest that contrast may result from automatically activated and operating nonconsciously accuracy motivation. Those explanations suggest that nonconscious processes are not entirely uncontrolled (see Glaser, 2003).

Coming back to the effects of suboptimal affective priming we argue that the assimilation effect can be modified under certain circumstances such as more salient (lasting longer) affective stimulation. Thus, the nature of nonconscious affective priming may be more complex that was originally assumed and may be mediated by automatic processes of implicit emotion regulation (Bargh and Williams, 2007). This way of thinking is supported by recent studies of Koole and colleagues (Koole and Jostmann, 2004; Koole and Rothermund, 2011a,b). They discuss the role of implicit emotion regulation and refer to it as counter-regulation. This line of studies shows that incongruency effect (that is defined in line with contrast effect definition presented above) appears when induced emotions are quite strong (affectively hot context) and there is a need to employ affect regulation).

Summing up, even when affect is elicited nonconsciously, some processes of (implicit) emotion regulation may appear automatically and modify the influence of affect on subsequent processes, as when we observe the contrast effect. This is one more argument for the substantial role of implicit processes in human life.

### Conflict of interest statement

The authors declare that the research was conducted in the absence of any commercial or financial relationships that could be construed as a potential conflict of interest.
